# Assessment of the Antioxidant Capacity of Commercial Coffee Using Conventional Optical and Chromatographic Methods and an Innovative Electrochemical DNA-Based Biosensor

**DOI:** 10.3390/bios13090840

**Published:** 2023-08-24

**Authors:** Stephanie L. Morais, Diana Rede, Maria João Ramalhosa, Manuela Correia, Marlene Santos, Cristina Delerue-Matos, Manuela M. Moreira, Cristina Soares, Maria Fátima Barroso

**Affiliations:** 1 REQUIMTE/LAQV, Instituto Superior de Engenharia do Porto, Instituto Politécnico do Porto, Rua Dr. António Bernardino de Almeida 431, 4249-015 Porto, Portugal; stephanielopesmorais@gmail.com (S.L.M.); dsgmr@isep.ipp.pt (D.R.); mjr@isep.ipp.pt (M.J.R.); mmb@isep.ipp.pt (M.C.); cmm@isep.ipp.pt (C.D.-M.); 2 Centro de Investigação em Saúde e Ambiente, Escola Superior de Saúde (CISA|ESS), Instituto Politécnico do Porto, Rua Dr. António Bernardino de Almeida 400, 4200-072 Porto, Portugal; mes@ess.ipp.pt

**Keywords:** coffee, bioactive compounds, DNA-based biosensor, HPLC-DAD, reactive oxygen species, total antioxidant capacity

## Abstract

As one of the most popular beverages in the world, coffee is a rich source of non-enzymatic bioactive compounds with antioxidant capacity. In this study, twelve commercial coffee beverages found in local Portuguese markets were assessed to determine their total phenolic and flavonoid contents, as well as their antioxidant capacity, by conventional optical procedures, namely, ferric reducing antioxidant power and DPPH-radical scavenging assay, and non-conventional procedures such as a homemade DNA-based biosensor against two reactive radicals: HO^•^ and H_2_O_2_. The innovative DNA-based biosensor comprised an adenine-rich oligonucleotide adsorbed onto a carbon paste electrode. This method detects the different peak intensities generated by square-wave voltammetry based on the partial damage to the adenine layer adsorbed on the electrode surface by the free radicals in the presence/absence of antioxidants. The DNA-based biosensor against H_2_O_2_ presented a higher DNA layer protection compared with HO^•^ in the presence of the reference gallic acid. Additionally, the phenolic profiles of the twelve coffee samples were assessed by HPLC-DAD, and the main contributors to the exhibited antioxidant capacity properties were caffeine, and chlorogenic, protocatechuic, neochlorogenic and gallic acids. The DNA-based sensor used provides reliable and fast measurements of antioxidant capacity, and is also cheap and easy to construct.

## 1. Introduction

Oxidative stress is commonly described as the imbalance between the production and accumulation of free radicals, such as oxygen reactive species (ROS), in our body’s cells and tissues and its ability to detoxify these reactive products [1,2]. Generated as metabolic by-products of the internal and external Fenton or Heiber Weiss reactions, hydrogen peroxide (H_2_O_2_), hydroxyl radicals (HO^•^), singlet oxygen (^1^O_2_), and superoxide radicals (O_2_^•−^) are well-known ROS with an important role in the apoptosis, differentiation, immunity, and protein phosphorylation processes, for example [1,2]. However, as they accumulate, these radicals can inflict damage on crucial structural macromolecules (e.g., proteins, lipids, and nucleic acids), as well as compromise the in vivo antioxidant defense mechanisms necessary to counteract the reactive nature of ROS. This oxidative damage has the potential to contribute to the development and progression of various diseases, including cancer, diabetes, metabolic disorders, cardiovascular diseases, Parkinson’s disease, Alzheimer’s disease, and other neurodegenerative disorders [1,2,3,4,5].

Studies have shown that our diet plays a major role in acquiring antioxidants to combat the reactive nature of free radicals [2]. To that end, numerous experiments have been conducted in different foods [6,7] and beverages [7,8,9,10,11,12] to determine which matrix exhibits a higher antioxidant activity to counteract and/or prevent further damage to the biological system. 

Coffee is a food product obtained from a tropical perennial tree of the *Coffea* genus and is one of the most popular beverages consumed worldwide [13]. With a revenue of USD 471.40 billion in 2022, the most popular coffee varieties are *Coffea arabica* (Arabica coffee) and *Coffea canephora* (Robusta coffee), representing 90% of the global coffee production [13,14,15]. Due to its flavor, aroma, and stimulant effects, drinking coffee has become a daily routine and social practice in many countries [3,16]. In Portugal, the total amount of coffee transactions in 2022 reached 590 million euros, with Arabica and Robusta coffee being the most consumed. 

Although coffee is typically associated with caffeine, it presents many bioactive compounds (caffeoylquinic, chlorogenic, ellagic, gallic, and tannic acids, esters of caffeic and ferulic acids with quinic acid, flavonoids such as catechin, epicatechin, and quercetin, among others) beneficial to human health when drunk in moderation [15,17]. The antioxidants present in coffee and coffee-like beverages can scavenge radicals and prevent the deleterious effects induced by them in the cells and tissues [18]. The latest research indicates that coffee may reduce oxidative stress and the risk of certain metabolic disorders [3,19,20,21] and neurodegenerative disorders [3,15,22], namely, Parkinson’s and Alzheimer’s [15], type 2 diabetes [3,15,20,23], and cardiovascular diseases [3,19,23,24], as well as slow the aging process [3]. Considering these health benefits, it becomes important to assess the composition and quality of coffee available in the market.

Analytical methodologies for total antioxidant capacity (TAC) assessment include spectrometry, chromatography, and electrochemical procedures [11]. The first two, although sensitive and specific, are normally expensive, time-consuming, and require qualified professionals [25]. Therefore, considering the electrochemical methods’ potential for miniaturization, low operation cost, short detection time, and ability to detect the electrochemical signal generated during the oxidative damage produced by HO^•^ and/or H_2_O_2_ in the presence/absence of antioxidant compounds in small volumes with high sensitivity and accuracy, they represent a useful tool for antioxidant measurement [11]. Electrochemical DNA-based biosensors are particularly suitable for TAC assessments since their electrochemical response is closer to the actual antioxidant activity produced in biological systems, i.e., it simulates the damage caused in vivo by ROS [11].

In this study, the antioxidant compounds found in twelve different coffee beverages from four brands commercially available in Portuguese markets were analyzed. Different methods, namely, the UV–Vis spectrometry analytical assays including ferric reducing antioxidant power (FRAP), total phenolic content (TPC) and total flavonoid content (TFC), 2,2-diphenyl-1-picryl-hydrazyl-hydrate-radical scavenging assay (DPPH-RSA), and high-performance liquid chromatography with diode array detection (HPLC-DAD) were used to assess and compare the antioxidant activity of the twelve coffee samples with the electrochemistry determined by a DNA-based biosensor against two ROS (H_2_O_2_ and HO^•^). These biosensors are based on the DNA-based materials (adenine or guanine) immobilization onto carbon paste electrodes (CPE) via adsorptive processes, which are then exposed to ROS and an antioxidant compound [11]. The electrochemical measurements were carried out by square-wave voltammetry (SWV) to estimate (by observing the decrease in the oxidation current of the DNA nucleobases compared with the intact DNA-based material’s electroactivity) the DNA damage induced by the ROS in question [11]. Accordingly, the protective effect of the antioxidants on the DNA-based material can also be estimated by analyzing the increase in the electrochemical current attributed to the antioxidants’ scavenging activity [11].

**Scheme 1:**
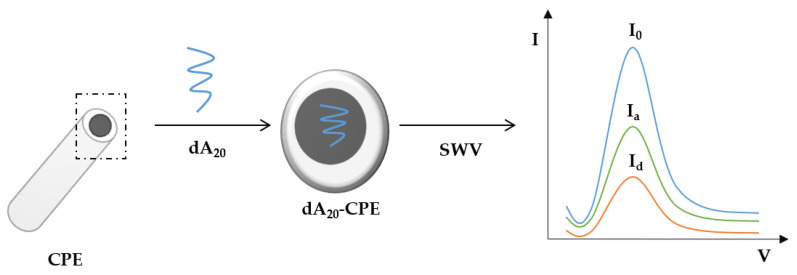
General scheme of the electrochemical DNA-based biosensor design. Electrochemical signal of the (I_0_) blank; Immerged dA_20_-CPE in the presence (I_a_) antioxidant and ROS species; and (I_d_) without the antioxidant.

The developed biosensor was constructed by immobilizing a 20-mer adenine oligonucleotide (dA_20_) on a homemade CPE. The experiments were structured in three steps (Scheme 1) consisting of (i) DNA (dA_20_) immobilization onto the CPE surface, (ii) measurement of the electrochemical signal through SWV and (iii) measurement of the electrochemical signal generated during the oxidative damage produced by HO^•^ or H_2_O_2_ in the presence/absence of an antioxidant compound.

## 2. Materials and Methods

### 2.1. Reagents and Solutions

The desalted deoxyadenylic acid oligonucleotide (dA_20_), Folin–Ciocalteau reagent, 2,2-diphenyl-1-picryl-hydrazyl-hydrate (DPPH), concentrated saline sodium phosphate EDTA (20× SSPE; 0.2 mol/L sodium phosphate, 2 mol/L NaCl, 0.02 mol L^−1^ EDTA), phosphate buffer (PBS) pH 7.4, and hydrogen peroxide (30%, *w*/*v*) were purchased from Sigma-Aldrich (Spain).

Gallic acid (GA), ascorbic acid (AA) and Epicatechin (EPIC) were obtained from Fluka, (Barcelona, Spain), and the graphite powder was from Ultracarbon (Spain). The different standards of phenolic compounds, namely, the phenolic acids (protocatechuic acid (99.63%), neochlorogenic acid (≥98%), caftaric acid (≥97%), 4-O-caffeyolquinic acid (≥98%), vanillic acid (≥97%), syringic acid (≥98%), *trans*-ferulic acid (≥99%), sinapic acid (≥99%), cinnamic acid (≥99%), caffeic acid (≥98%), *p*-coumaric acid (≥98%), chlorogenic acid (>95%), 3,5-di-O-caffeyolquinic acid (≥95%), ellagic acid (≥95%), and 3,4-di-O-caffeyolquinic acid (≥90%)); flavonoids ((+)-catechin (≥98%), naringin (≥95%), naringenin (98%), rutin hydrate (≥94%), quercetin (95%), kaempferol (≥98%), myricetin (≥96%), quercitrin (≥98.5%), quercetin-3-O-galactoside (≥97%), quercetin-3-O-glucopyranoside (≥99%), kaempferol-3-O-rutinoside (≥98%), kaempferol-3-O-glucoside (≥95%), isorhamnetin-3-O-glucoside (≥98%), isorhamnetin-3-O-rutinoside (≥99%), tiliroside (≥98%), apigenin (≥95%), chrysin (97%)); chalcones (phloridzin dehydrate (99%) and phloretin (≥98.5%)); and stilbenoids (resveratrol (≥99%), *trans*-polydatin (≥98%), *trans*-e-viniferin (≥90%), and caffeine (99%)) were obtained from Sigma-Aldrich (Madrid, Spain) and Extrasynhtese (Genay, France). Their individual stock solutions were prepared in methanol at concentration levels ranging from 1 to 5 g/L and stored at −20 °C. Analytical grade methanol and the remaining reagents were acquired from Merck (Darmstadt, Germany).

Stock solutions of 1 g/L dA_20_ were stored at 4 °C and diluted with 2× SSPE buffer solution (from a 20× SSPE solution) prior to use. Fenton solution (to produce the hydroxyl radical) was prepared by mixing Fe^2+^:EDTA:H_2_O_2_ (1 μmol/L:2 μmol/L:40 μmol/L) in the molar ratio of 1:2:40. All solutions were prepared with ultra-pure water produced with a Simplicity 185 system manufactured by Millipore (Darmstadt, Germany).

### 2.2. Apparatus and Electrode 

A Multi-Mode Microplate Reader (BioTek Instruments, Winooski, VT, USA) was employed to carry out the conventional optical analytical methods, such as TPC, TFC, FRAP and DPPH.

The HPLC analyses were carried out in a Shimadzu HPLC Prominence system (Kyoto, Japan) equipped with a LC-20AD pump, a DGU-20AS degasser, a CTO-10AS VP column oven, a SIL-20A HT autosampler, and an SPD-M20A photodiode array detector.

SWV was performed in an Autolab PSTAT 10 controlled by GPES software, version 4.8 (EcoChemie, Utrecht, The Netherlands). A conventional three-electrode cell was used, and included a homemade CPE (2 mm in diameter; electroactive area of 0.047 cm^2^) as a working electrode, a platinum wire-counter electrode and an Ag|AgCl|KCl sat reference electrode to which all potentials are referred. The CPE was prepared by mixing 1.8 g of paraffin oil as pasting liquid with 5 g of spectroscopic grade graphite powder. The unmodified carbon paste (CP) was introduced into the well of a Teflon electrode body provided by a stainless-steel piston. The surface was smoothed against a plain white paper while a slight manual pressure was applied to the piston. Since CP oxidizes when exposed to air, for each analysis a new CPE surface was freshly prepared [6].

### 2.3. Samples

Twelve different coffee samples from five commercial brands (Table 1) were acquired in local food markets in Porto, Portugal. The ground coffee samples were prepared with boiling water (100 °C), whereas the capsule coffee samples were prepared with a coffee machine.

### 2.4. DNA-Based Biosensor Procedure

The experiments were conducted in three steps (Scheme 1): (i) DNA immobilization; (ii) irreversible damage of oligonucleotide (dA_20_) by immersion of the dA_20_-CPE on either the HO^•^ (Fenton solution) (Equation (1)) or in H_2_O_2_ radical in the absence/presence of antioxidants or commercial coffee samples (Equations (2) and (3)); and (iii) detection and measurement of the peak current (I_p_) of dA_20_ in a PBS solution at pH 7.4. I_p_ is the peak current obtained under SWV. When SWV is applied to the DNA-based biosensor over the potential range of 1.0 to 1.4 V, a peak potential (E_p_) appears at around 1.12 V. This E_p_ corresponds to the adenine electro-oxidation. The maximum I_p_ is obtained when the DNA-based biosensor (without any type of interaction) is inserted in PBS solution, and the registered current is called I_o_ (the blank signal). All subsequent measurements are related to I_o_.

DNA immobilization was performed by dry adsorption, placing a 4 mL droplet of dA_20_ (100 mg/L) [26] in 2× SSPE solution on the electrode surface, taking care to cover the whole surface, and evaporating it to dryness under a stream of nitrogen. To obtain the blank signal (I_0_) expressed as the dA_20_ signal (without damage either antioxidant effect), a freshly prepared dA_20_-CPE was immersed in PBS pH 7.4 and the SWV recorded. DNA damage was carried out by immersing the DNA-based CPE in a freshly prepared Fenton solution or H_2_O_2_ solution in the absence or presence of the standard antioxidant or beverage samples in 2× SSPE buffer.

Fe^2+^ + H_2_O_2_ → Fe^3+^ + OH^−^ + OH^•^ (1)

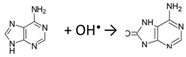
(2)

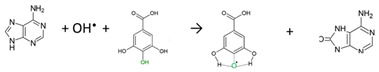
(3)

After 30 s of reaction time, the DNA-based CPE was washed with water and immediately immersed in PBS (pH 7.4). SWV (frequency = 25 Hz; step potential = 5.1 mV and amplitude = 20 mV) [12] was then conducted between +0.7 and +1.5 V, and the oxidation I_p_ of dA_20_ obtained was recorded as the detection signal. For the electrochemical studies, it was considered that the maximum signal current obtained was for the dA_20_ electrochemical signal without damage to either antioxidant effect (Scheme 1). Each coffee sample was measured in triplicate. The validated calibration curve was used to determine the TAC. The results are expressed as mg GA equivalents (GAE) per liter of beverage. 

### 2.5. Conventional Optical Analytical Methods

#### 2.5.1. Total Phenolic Content

TPC values were determined by a colorimetric assay based on Folin–Ciocalteau reagent. Calibration curves were obtained using GA according to Barroso et al. [11]. The experiment was carried out in triplicate, and the results are expressed as mg GAE per liter of beverage.

#### 2.5.2. Total Flavonoid Content

TFC was determined by a colorimetric assay based on the formation of a flavonoid–aluminum compound as described previously [11]. Calibration curves were prepared with EPIC solutions, and results are expressed as milligrams of epicatechin equivalents (EPICE) per liter of beverage. The experiment was carried out in triplicate. 

#### 2.5.3. Ferric Reducing Antioxidant Power (FRAP) Assay

The FRAP method was carried out according to [11]. The calibration curve was prepared with AA. The samples were analyzed in triplicate and expressed as mg ascorbic acid equivalents (AAE) per liter of beverage.

#### 2.5.4. 2,2-Diphenyl-1-Picryl-Hydrazyl-Hydrate-Radical Scavenging Assay

DPPH-RSA of samples was determined spectrophotometrically at 517 nm, as reported by [11]. The calibration curve was prepared with Trolox solutions and the results are expressed as Trolox equivalents (TE) per liter of beverage. The experiment was carried out in triplicate.

### 2.6. HPLC-DAD

The phenolic profiles of coffee and coffee substitutes available in the Portuguese market were analyzed according to the method described by Moreira et al. [26]. Chromatographic analyses were performed using a Shimadzu HPLC system, and polyphenols separation was achieved on a Gemini C18 column (250 × 4.6 mm, 5 μm) from Phenomenex (Lisbon, Portugal) thermostatized at 25 °C. The solvent system used, pumped at a flow rate of 1 mL/min, was methanol (eluent A) and water (eluent B), both acidulated with formic acid (0.1%). Chromatograms were registered at 280, 320 and 360 nm depending on the maximum absorption of the phenolic compound identified. Before injection, coffee samples were 2 times diluted and filtered through a 0.22 μm PTFE hydrophilic syringe filter. The quantification of the phenolic compounds was based on calibration curves of the pure standards, and results are expressed as mg of compound per L of coffee.

### 2.7. Statistical Analysis

Statistical analysis was performed using IBM SPSS v. 28 (IBM Corp., Armonk, NY, USA). Data of all analysis are expressed as mean ± standard error. The Kolmogorov–Smirnov test was used to evaluate data normality. The one-way ANOVA was used to assess significant differences between samples, followed by Tukey’s HSD post hoc test to make pairwise comparisons among means. When data were not normally distributed, the non-parametric Kruskal–Wallis test was used. The level of significance for all hypothesis tests (*p*) was 0.05. Pearson correlation tests were used to ascertain the existence of linear relationships between the different parameters analyzed.

## 3. Results and Discussion

**Scheme 2:**
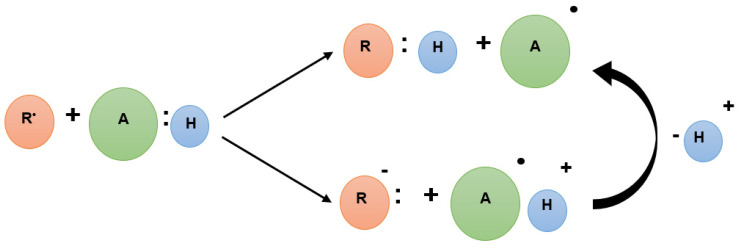
Mechanisms of action of the Hydrogen Atom Transfer (HAT) and Single Electron Transfer (SET) reactions form the antioxidant activity (adapted from [2]).

Antioxidants play an essential role in the defense mechanism against the redox imbalances that originate oxidative stress and, consequently, irreversible modifications to a cell’s structural compounds by (i) delaying/inhibiting the production of free radicals; (ii) scavenging free radicals; (iii) altering free radicals into less noxious compounds; (iv) delaying the development of secondary reactive species; (v) interrupting the propagation of chain-breaking antioxidants; or (vi) enhancing the endogenous antioxidant defense mechanism through the synergy with other antioxidants [27]. All the above fundamentally result from the transfer of electrons to free radicals, which inhibits or slows the oxidative process [27]. Generally, antioxidants react to free radicals by either a hydrogen atom transfer mechanism, a single electron transfer mechanism, or a combination of both (Scheme 2) [2]. 

An ideal antioxidant should be easily absorbed/dissolvable and remove ROS in adequate physiological concentrations [27]. Phenols are a large group of chemical compounds (which possess at least one HO^•^ group attached to an aromatic ring) with diverse bioactivities that depend on their chemical structure to act as oxidant scavengers [27]. Thus, the evaluation of the antioxidant properties of phenolic compounds is of interest. However, as no single assay is capable of accurately determining the antioxidant profile of any given sample, a series of procedures must be utilized. In this study, a DNA-based biosensor was employed for the TAC assessment of twelve commercial coffees. The electrochemical sensor evaluated the TAC by reproducing an in vivo assay through the exposure of the 20-mer single stranded DNA (ssDNA) adenine layer to the ROS and GA antioxidant [9]. The obtained results were validated by comparing them with those from the well-described optical methods, namely, TPC, TFC, FRAP and DPPH, as well as by correlation with the phenolic profile determined by HPLC-DAD.

### 3.1. Electrochemical DNA-Based Biosensor 

The physical and chemical composition of a transducer can alter the peak potential of the ssDNA oligonucleotide of adenines immobilized onto its surface. Previous studies determined that the electro-oxidation of an adenine ssDNA sequence immobilized onto a CPE by an adsorptive or electrodeposition leads to an irreversible electrochemical oxidative process with a peak potential of +1.20 V vs. AgCl/Ag [9]. Furthermore, the analytical performance of the electrochemical biosensor is closely linked with the working electrode’s surface immobilization and coverage by the recognition element as it can diminish the charge transfer rate on the electrode’s surface. This can be regulated by optimizing the ratio between the recognition element (in this case, the dA_20_) and the working electrode’s surface area [11,28].

The electrochemical oxidation peak is also extremely dependent on DNA-based materials’ flexibility and availability on the transducer surface [11]. Therefore, ssDNA was chosen for the TAC assessment instead of double-stranded DNA (dsDNA) as all the bases from the ssDNA should be more available to the electrode surface and, thus, easily electro-oxidized, producing higher peaks [11].

According to the literature, HO^•^ is a highly reactive oxidant with a small half-life (10^−9^ s), which makes it an extremely dangerous free radical for biological systems like the human body [11].

**Figure 1:**
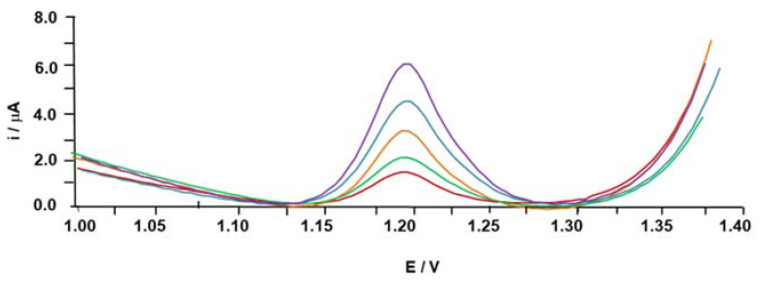
Electrochemical responses of the DNA-based biosensor when immersed in the absence (intact DNA) and presence of the two reactive oxygen species and in the presence and absence of the gallic acid antioxidant. The intact DNA signal is shown in purple, gallic acid in the presence of the H_2_O_2_ solution in blue, H_2_O_2_ solution in green, HO^•^ solution immersed in the gallic acid in orange and the HO^•^ solution without the antioxidant compound in red.

SWV was the voltammetric technique used to study the protective effect of GA (against the HO^•^ and H_2_O_2_ solutions) on the i_p_ of the DNA-based biosensor. For this analysis, 3.0 mg/L of the GA antioxidant was applied to the system. Figure 1 displays the DNA signals before (undamaged DNA) and after the immersion of the dA_20_-CPE biosensor in the oxidative system containing the HO^•^ and H_2_O_2_ oxidants in the presence and absence of the GA antioxidant compound.

GA is a major plant compound with a relatively simple molecular structure (trihydroxy phenol), and is recognized for its antioxidant activity [27]. For instance, GA has demonstrated cardiovascular-protective effects in hypertension subjects and beneficial effects on induced gastric mucosal damage by preventing oxidative stress and decreasing apoptosis in gastric mucosal cells [27].

As shown in Figure 1, the maximum DNA signal (5.7 μA) was obtained before the immersion of the DNA-based electrode in the reactive species (HO^•^ and H_2_O_2_) solutions, i.e., for the undamaged dA_20_, since in their absence, the adenines were accessible for electrochemical oxidation. On the other hand, both ROS presented lower i_p_ values than the intact dA_20_ signal (i_p_ = 1.6 μA and 2.2 μA for the HO^•^ and H_2_O_2_ radicals, respectively). These results confirm the ROS’ ability to induce oxidative damage on biomolecules and, as expected, the HO^•^ radical induced the highest DNA lesion; dA_20_-CPE/HO^•^ had the lowest i_p_ of the two ROS, resulting in 72% oxidative damage in comparison with dA_20_-CPE/H_2_O_2_, which had 61% damage. 

Since HO^•^ is the more reactive ROS, antioxidants inevitably present a higher ability to counteract the H_2_O_2_ than the HO^•^ radical. Potent antioxidants with a high scavenging ability are required to counteract ROS damage and promote biomolecule protection [11]. GA is an antioxidant found in coffee with an active protective effect against ROS. The effect of the GA scavenger on DNA protection was also studied, and a protective effect to the dA_20_-CPE sensor of 55% and 95% for the HO^•^ and H_2_O_2_ oxidants, respectively, was electrochemically registered (Figure 1). As predicted, the lowest scavenger activity was obtained with the HO^•^ radical.

The calibration curves were recorded for the i_p_ obtained after immersion of the DNA-based sensor in HO^•^ and H_2_O_2_ radicals with increasing concentrations of GA ranging from 1.50 to 6.00 mg/L in the reactive system.

A linear range was obtained for the varying GA concentrations in the HO^•^ reactive system and is expressed by the mathematical equation: i_p_ (μA) = 0.97 ± 0.09 [GAE mg/L] + 1.00 ± 0.04 r^2^ = 0.9984; *n* = 4. When H_2_O_2_ was used as the dA_20_ oxidant, a linear relationship (r^2^ = 0.9844; *n* = 5) was obtained for the GA concentration varying from 1.50 to 6.00 mg/L, with slope and intercept values of 1.04 ± 0.03 (μA/GAE mg/L) and 0.64 ± 0.0.08 (μA), respectively. 

In both systems, the intensity of the i_p_ improved with the increasing concentration of GA. The lowest scavenger activity was found when the dA_20_-CPE was immersed in the HO^•^ oxidant with a concentration of 1.50 mg/L of GA. The limit of detection (LOD) was calculated according to Miller and Miller [29]. The LOD values were 2.75 and 2.32 mg GAE/L when HO^•^ or H_2_O_2_ were used as ROS, respectively. 

The precision of the results was studied by intra- and inter-day (1 week) assays at a GA concentration of 3 mg/L and was determined by calculating relative standard deviations (RSDs). For the intra-day studies, no significant differences were found between intra-day and inter-day experiments, with RSD values ranging between 5.3% and 8.3%, indicating the high precision of the electrochemical device.

### 3.2. TAC Assessment 

Green coffee beans are in themselves a rich source of bioactive compounds with different free radical characteristics and antioxidant activity [2]. Nonetheless, the coffee bean roasting process produces a series of physical, chemical, and sensory changes to the coffee’s composition, leading to changes in phenol compounds that may contribute to increasing the antioxidant activity [2,30]. For example, the green Arabica and Robusta coffees have average TPC levels of 106.5 and 129.0 mg GAE/100 g, respectively, while the roasted 100% Arabica and 100% Robusta contain 221.8 and 226.9 mg of GAE/100 g, respectively [31]. 

**Figure 2:**
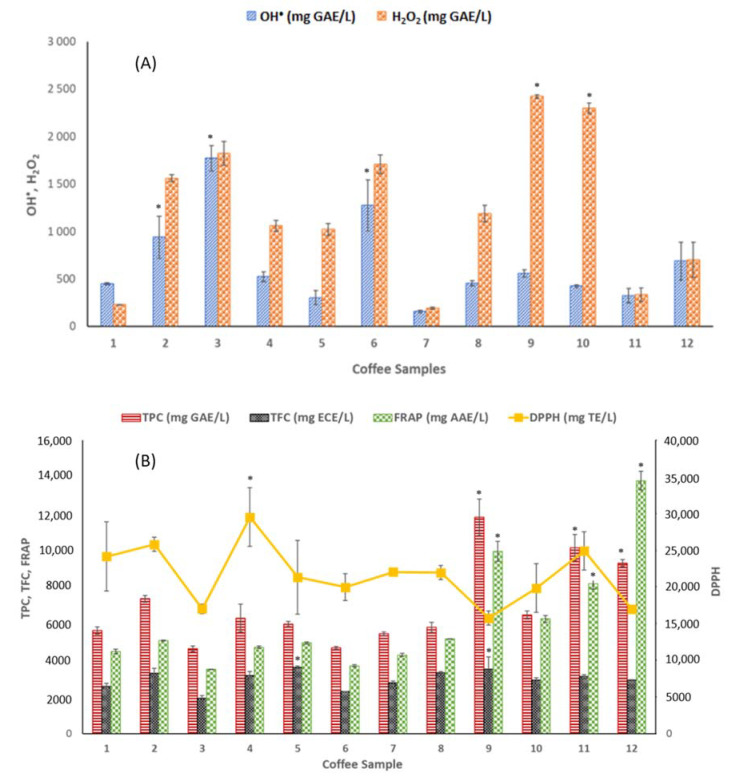
Total antioxidant capacity of commercial coffee samples measured by (**A**) DNA-based biosensor and (**B**) optical methods. * Represents the maximum values yielded by each method, which are significantly different from the others (*p* < 0.05).

As no single test of antioxidant level is sufficient, a “battery” comprising multiple techniques employing distinct modes of action and foundations is used. Therefore, the antioxidant capacities of hydrophilic coffee components (e.g., caffeine, chlorogenic acid and melanoidins) and hydrophobic compounds (such as cafestol, kahweol and trigonelline) against the ROS have been investigated using different assays [2]. The full antioxidant status of the twelve commercial coffee samples was evaluated by three different methodologies: an electrochemical DNA-based biosensor, the conventional optical methods (TPC, TFC, FRAP and DPPH), and the chromatographic (HPLC) methods. Figure 2A and Table S1 present the results obtained from the analysis of the DNA-based biosensor and optical procedures, and Table S2 describes the main phenolic compounds identified in the coffee samples by the HPLC-DAD assessment. 

#### 3.2.1. DNA-Based Biosensor

TAC values varying from 155.9 to 1771 mg GAE/L (when immersed in the HO^•^ radical) and 193.43 to 2422.60 mg GAE/L (for the H_2_O_2_ solution) were measured utilizing the electrochemical DNA-based biosensor developed. In general, the TAC levels were higher when H_2_O_2_ was used as the radical to the dA_20_ when compared with HO^•^. Sample 1 (intensity 9, Arabica and Robusta coffee capsule) was the only commercial coffee with a lower scavenging capacity for the H_2_O_2_ than the HO^•^ oxidant. 

A coffee’s physicochemical composition is greatly influenced by various parameters (e.g., the coffee plant species, geographical origin, growth conditions, agricultural practices, processing, and storage, among others) [15,32]. Moreover, it has been proved that even the same coffee species can present different amounts of phenolic compounds when grown in distinct geographical regions [17,33,34]. For instance, Catelani et al. [33] and Baeza et al. [34] demonstrated that the Timorese and Ethiopian Arabica coffees present higher concentrations of polyphenols than the Colombian and Brazilian Arabica coffees [33,34]. Zhu et al. [15] further investigated this subject and determined that the average chemical composition of coffees presented decreasing content of phenolic compounds in the following order of geographical origin: Indonesia > Honduras > Ethiopia > Guatemala > China > Kenya > Colombia > Brazil [15]. 

Furthermore, different coffee species also present several TAC variations depending on their brewing [31] and roasting process [30]. Hasbullah and Umiyati [30] and Olechno et al. [31] determined the effect of different roasting and brewing methods on the TPC of Arabica and Robusta coffees. Briefly, Hasbullah and Umiyati [30] reported that the difference in the roasting levels (light, medium and dark) affects the antioxidant activity and TPC of Arabica and Robusta coffee. Light Arabica coffee normally displays a higher antioxidant activity and TPC [30]. Meanwhile, Olechno et al. [31], concluded that different brewing methods have a considerable influence on the TPC analysis. Furthermore, green (Arabica and Robusta) coffee has a lower TPC value than roasted (Arabica and Robusta) coffee and higher TPC values were found in coffees sold in transparent packaging in comparison to those in opaque packaging [31].

Regarding the HO^•^ reactive system (Table S1, Figure 2A), sample 7 (intensity 8, Arabica and Robusta coffee capsule) presented the lowest TAC value (155.9 mg GAE/L). In contrast, sample 3 (intensity 7, Arabica and Robusta decaffeinated coffee capsule) reported the highest (1771 mg GAE/L). Both (the highest and lowest recorded) values derive from an Arabica and Robusta blend coffee sample. In fact, the TAC assessment levels increased as followed: sample 7 (155.9 mg GAE/L; intensity 8, Arabica and Robusta coffee capsule) < sample 5 (303.3 mg GAE/L; intensity 10, Arabica and Robusta coffee capsule) < sample 11 (322.16 mg GAE/L; intensity 9, Arabica decaffeinated coffee capsule) < sample 10 (425.3 mg GAE/L; Arabica ground coffee) < sample 1 (448.4 mg GAE/L; intensity 9, Arabica and Robusta coffee capsule) < sample 8 (454.5 mg GAE/L; intensity 5, Arabica and Robusta coffee capsule) < sample 4 (523.2 mg GAE/L; intensity 13) < sample 9 (559.5 mg GAE/L; intensity 7, Arabica and Robusta coffee capsule) < sample 12 (688.1 mg GAE/L; Arabica and Robusta instant coffee) < sample 2 (939.4 mg GAE/L; intensity 10, Arabica and Robusta coffee capsule) < sample 6 (1275 mg GAE/L; intensity 9, Arabica coffee capsule) < sample 3 (1771 mg GAE/L; intensity 7, Arabica and Robusta decaffeinated coffee capsule).

Coffee with a high caffeine content is reported to possess a higher antioxidant activity [32,35,36,37,38]. Furthermore, the coffee’s chemical composition is greatly influenced by numerous factors, such as its processing [15,32]. In this regard, the Arabica and Robusta mix coffee samples (samples 1–3, 5, 7, 9 and 12) presented varied concentrations of TAC. Sample 3, the only mixed decaffeinated coffee, registered the highest TAC value, with an average TAC of 1771 mg GAE/L, while the mixed caffeinated sample with the highest antioxidant activity was sample 2, with an average TAC of 939.4 mg GAE/L, followed by samples 12, 9, 1, 5 and 7 (588.1–155.9 mg GAE/L). As for the 100% Arabica coffees (samples 6, 8 10 and 11 a similar TAC range variation was also observed. The caffeinated sample 6 (1275 mg GAE/L) obtained the highest TAC value, followed by samples 8 and 10 (454.47 and 425.26 mg GAE/L, respectively). The decaffeinated coffee sample 11 (322.2 mg GAE/L) presented the lowest TAC value.

Sample 4 is a mixed power coffee that contains two times more caffeine (intensity 13). Nevertheless, sample 4 presents a lower TAC value (523.20 mg GAE/L) than expected. In comparison to the other blended samples (samples 1–3, 5, 7, and 12), the power coffee has an intermediate TAC level, i.e., sample 4 has a higher damage protection potential than sample 1, 5 and 7 but lower than that of samples 2, 3 and 12. The mixture of the blended coffee with the guarana and ginseng seems to only have a slight influence on the antioxidant scavenging activity. 

Regarding samples 3 and 11, which are decaffeinated coffees, the decaffeinated Arabica + Robusta mixture (sample 3) presents a much higher TAC value (1771.00 mg GAE/L) than the 100% Arabica decaffeinated sample 11 (322.2 mg GAE/L). In this case, the decaffeination process seems to have an influence on the different coffee compositions; however, with our current results, a linear correlation of its effect in the TAC concentration is not visible. On the other hand, instant coffee (sample 12) presents a TAC value isolated from all other samples.

Analyzing the present data (Table S1), only two coffees (samples 1 and 5) measured against the HO^•^ radical presented significant differences (*p* < 0.05). In terms of TAC values (evaluated by the DNA-based biosensor against the HO^•^ radical), the studied coffee samples are considered dissimilar. 

Concerning the TAC assessment against the H_2_O_2_ oxidant, the highest TAC value (2422.6 mg GAE/L) was found for sample 9 (intensity 7 coffee capsule) and the lowest (193.4 mg GAE/L) for sample 7 (intensity 8 coffee capsule). In this system, the TAC levels increased in the following order: sample 7 (193.4 mg GAE/L) < sample 1 (227.9 mg GAE/L) < sample 11 (335.0 mg GAE/L) < sample 12 (699.4 mg GAE/L) < sample 5 (1021 mg GAE/L) < sample 4 (1060 mg GAE/L) < sample 8 (1187 mg GAE/L) < sample 2 (1562 mg GAE/L) < sample 6 (1708 mg GAE/L) < sample 3 (1824 mg GAE/L) < sample 10 (2297.60 mg GAE/L) < sample 9 (2422 mg GAE/L). 

The Arabica and Robusta mix coffee samples (samples 1–3, 5, 7, 9 and 12) presented an ample range in TAC values, having both the highest (2422 mg GAE/L) and lowest (193.4 mg GAE/L) values for the H_2_O_2_ studies. As for the HO^•^ system, samples 2, 1 and 7 presented decreasing TAC values in accordance with their intensity (intensity from 10 to 8). Samples 5 and 9 possessed, approximately, three and four times the value registered by the HO^•^ system, while samples 3 and 12 had similar intensities to the HO^•^ system. Sample 1 was the only coffee simply presenting a lower TAC value compared with the HO^•^ oxidant. 

As for the 100% Arabica samples (samples 6, 8, 10 and 11), sample 11 attained the lowest value (335.00 mg GAE/L). In contrast, sample 10 had the highest TAC value (2297.6 mg GAE/L), followed by sample 6 and 8 (1709 and 118 mg GAE/L, respectively). The mixed sample 4 presents approximately two times the registered TAC value from the HO^•^ system. Nonetheless, it still presents a lower TAC than predicted. 

Similar to the values obtained for the HO^•^ system, the TAC value was higher in the decaffeinated coffee consisting of the Arabica and Robusta mixture (sample 3; 1824 mg GAE/L) than in the decaffeinated coffee consisting only of Arabica (sample 11; 335.0 mg GAE/L). The instant coffee (sample 12) attained a similar value to the one from the HO^•^ reactive system. Sample 12 presented the third lowest activity (699.36 mg GAE/L) against the H_2_O_2_ radical.

Analyzing these values, sample 4 and 5 were the only two coffees (measured against the H_2_O_2_ radical) which presented a statistical difference (*p* < 0.05). As in the HO^•^ reactive system, these coffee samples are considered statistically different.

Overall, the high-intensity blended coffee samples exhibit a higher antioxidant capacity than the 100% Arabica samples. The presence of the Arabica and Robusta coffee species appears to have a positive protection effect against the HO^•^ and H_2_O_2_ radicals. In both systems, sample 7 presents the lowest TAC level. 

The statistical analysis for the HO^•^ radical revealed the existence of five statistically distinct groups. The first group consists of low TAC value coffees, including samples 1, 4, 5, 7–11, with TAC values ranging from 155.9 to 559.5 mg GAE/L. The next group is formed by samples 1, 2, 4, 8, 9 and 12, followed by the group comprising samples 1, 2, 4, 5, 8, 9 and 12. The fourth group included samples 2 and 6. Sample 6 (with a TAC of 1275 mg GAE/L) defines both the fourth and fifth statistical groups. 

Comparing the TAC data acquired from the DNA-based biosensor measurements against the two ROS in this study, it can be observed that the hierarchy of TAC values is to some degree similar. The statistical Pearson correlations (Table 2) indicate that a moderate correlation (0.4530) was observed between the TAC values for both biosensors. In fact, even with the substantial differences that exist in the mechanistic reaction involving DNA, ROS and antioxidants (the ROS action, e.g., HO^•^ is stronger in comparison with the H_2_O_2_ damager; the oxidant nature, i.e., HO^•^ is a free radical with an unpaired electron, while H_2_O_2_ is an intermediate oxidant; the differences in the ROS and antioxidant kinetic and chemical reaction pathways; and the different ROS interactions due to the sample compositions) a statistical correlation was still obtained [11].

The statistical analysis for H_2_O_2_ also signaled the presence of five statistical groups. The first group comprises the lowest TAC value samples (samples 1, 7 and 11), followed by the group consisting of samples 2, 3 and 6, with TAC values ranging from 1562 to 1709 mg GAE/L. The third group contains sample 4, sample 5 and sample 8, with TAC values ranging from 1021 to 1187 mg GAE/L. A group is formed by samples 5 (TAC = 1021 mg GAE/L) and 12 (TAC = 699 mg GAE/L). The last group includes the highest TAC value samples, sample 9 (2422 mg GAE/L) and sample 10 (2297 mg GAE/L).

In Carlsen et al. [7] the antioxidant content of thirteen beverages, including red wine, green and black tea, coffee, and fruit and vegetal juice, were analyzed. The expresso coffee registered the highest antioxidant content (14.20 mmol/100 g) of all the examined products, followed by red wine and boiled and filtered coffee beverages with the same concentration (2.50 mmol/100 g). The green and black tea infusions, which are known for their rich composition in bioactive compounds, presented an antioxidant content of 1.50 and 1.00 mmol/1000 g, respectively [7]. In Barroso et al. [11], a dA_20_-CPE electrochemical biosensor to measure the TAC of plant infusions (teas) was also employed. The highest recorded TAC value for the HO^•^ radical (228.75 mg AAE/L) and H_2_O_2_ oxidant (1279.98 mg AAE/L) corresponded to the green and peppermint tea infusions. Compared with the results obtained from the coffee TAC assessment (Table S1), tea infusions have an inferior protection potential than the commonly available coffees in the markets. Furthermore, Garcia and collaborators created a DNA-based biosensor using rutin as standard with an LOD of 12 μM (7.33 mg rutin equivalents/L) by increasing electrochemical currents with carbon nanotubes [39].

#### 3.2.2. Optical Methods

Regarding the optical procedures (Figure 2B and Table S1), TPC and TFC assays for all twelve coffee samples were performed. TPC was assessed using the Folin–Ciocalteau assay, whose basic mechanism is an oxidation/reduction reaction and can, therefore, be applied as a metric for antioxidant activities [32]. Since flavonoids are a group of phenolic compounds with antioxidant activities, TFC was assessed as an additional TAC indicator. The antioxidant activity of the analyzed coffee substitute brews was measured using FRAP and DPPH assays. 

Considering TPC, sample 9 (Arabica and Robusta instant coffee) contained, by far, the highest content of phenolic compounds (118,079 mg GAE/L). In contrast, sample 3 (intensity 7, Arabica and Robusta decaffeinated coffee capsule) presented the lowest phenolic content (4612 mg GAE/L). The phenolic values for the other samples ranged from 4670 to 10,149 mg GAE/L, for sample 6 (intensity 9, Arabica coffee capsule) and sample 11 (intensity 9, Arabica decaffeinated coffee capsule), respectively. All the coffee samples tested in the present study show higher TPC contents than those reported by Olechno et al. [31] and Chávez et al. [40]. 

Concerning the TFC, sample 5 (intensity 10, Arabica and Robusta coffee capsule) presented the highest content of total flavonoids (3588 mg ECE/L), followed by sample 9 (intensity 7, Arabica and Robusta coffee capsule) and sample 8 (intensity 5, Arabica coffee capsule) (3504 and 3304 mg ECE/L, respectively). On the other hand, sample 3 (intensity 7, Arabica and Robusta decaffeinated coffee capsule) exhibited the lowest flavonoid content (1940 mg ECE/L). 

**Figure 3:**
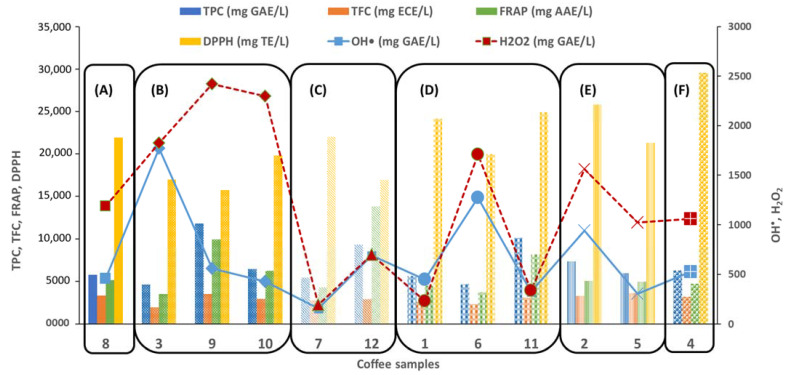
Total antioxidant capacity of commercial coffee samples measured by DNA-based biosensor and optical methods and compared with the coffee intensity: (**A**) 5; (**B**) 7; (**C**) 8; (**D**) 9; (**E**) 10; (**F**) 13.

**Figure 4:**
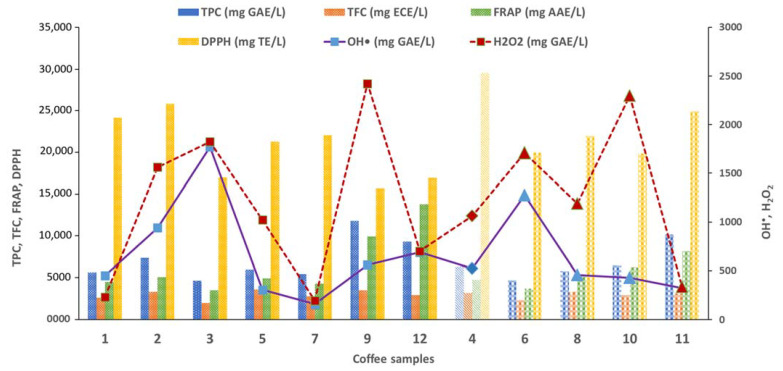
Total antioxidant capacity of commercial coffee samples measured by DNA-based biosensor and optical methods and compared with the type of coffee mixtures: Arabica + Robusta; blend coffee + guarana + ginseng; 100% Arabica.

The antioxidant activity was also assessed by FRAP and DPPH assays. Sample 3 showed the lowest FRAP value (3493 mg AAE/L), while sample 12 presented the highest (13,805 mg AAE/L). For the DPPH-RSA assay, sample 4 and 9 registered the highest (29,540 mg TE/L) and the lowest (15,725 mg TE/L) ability, respectively, to scavenge DPPH radicals. According to Yashin et al. [41], expresso coffee possesses slightly higher FRAP levels than instant coffees. Nevertheless, in our study, sample 12, the instant coffee sample, presented the highest TPC and FRAP values. Figure 3 shows the correlation between the TAC values and the coffee intensity, while Figure 4 displays the relation between the TAC values and the type of coffee mixtures. Pearson correlation tests were also conducted to determine the linear relationships between the different methods (Table 2). The highest linear correlation was found between FRAP and TPC (r = 0.8140, *p* < 0.01). Other significantly high correlations (*p* < 0.01) were observed between the TPC and TFC (0.5380, *p* < 0.01) and between TFC and the DNA-based biosensor against the HO^•^ radical (0.6150, *p* < 0.01).

#### 3.2.3. Phenolic Profile of Coffee Samples by HPLC-DAD

Phenolic compounds can be split into two main groups: (i) the phenolic acids, comprising hydroxylated derivatives of benzoic and cinnamic acids; and (ii) flavonoids, which include flavanols, flavones, flavanones, flavanols, anthocyanins and isoflavone [6,27].

**Figure 5:**
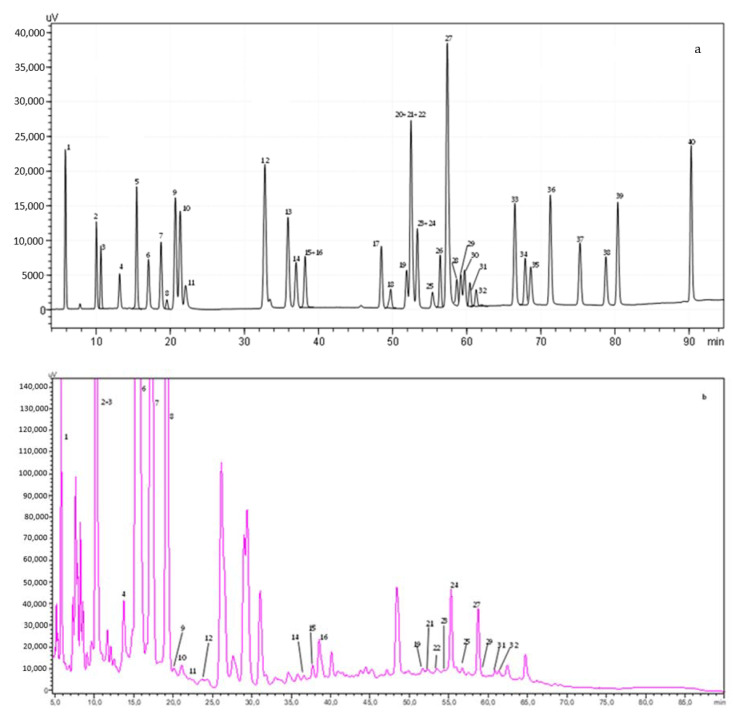
HPLC chromatogram acquired at 280 nm for (**a**) polyphenol standard mixture of 5 mg/L and coffee sample I (**b**). Peak identification: (1) gallic acid, (2) protocatechuic acid, (3) neochlorogenic acid, (4) (+)-catechin, (5) caftaric acid, (6) caffeine, (7) chlorogenic acid, (8) 4-O-caffeyolquinic acid, (9) vanillic acid, (10) caffeic acid, (11) syringic acid, (12) (−)-epicatechin, (13) p-coumaric acid, (14) trans-ferulic acid, (15) sinapic acid, (16) trans-polydatin, (17) naringin, (18) 3,5-di-caffeoylquinic acid, (19) quercetin-3-O-galactoside, (20) resveratrol, (21) quercetin-3-O-glucopyranoside, (22) rutin, (23) phloridzin, (24) ellagic acid, (25) 3,4-di-O-caffeoylquinic acid; (26) myricetin, (27) cinnamic acid, (28) quercitrin, (29) kaempferol-3-O-glucoside, (30) isorhamnetin-3-O-glucoside, (31) kaempferol-3-O-rutinoside, (32) isorhamnetin-3-O-rutinoside, (33) naringenin, (34) trans-epsilon viniferin, (35) quercetin, (36) phloretin, (37) tiliroside, (38) kaempferol, (39) apigenin, and (40) chrysin.

The phenolic compounds contributing to the samples’ antioxidant activity were identified and quantified by HPLC-DAD. Data were obtained by comparing the authentic standards’ retention time and the UV spectra from the resulting calibration curves. Figure 5 shows the chromatograms of the solution containing all 40 phenolic compounds and an example of the analyzed commercial coffee samples. Table S2 presents the amount of each compound determined in the analyzed coffees.

A total of 27 phenolic compounds were identified and/or quantified in the commercial coffee samples, with total amounts ranging from 733 to 4600 mg/L, corresponding to samples 2 and 9, respectively (Figure 5). Compounds belonging to the phenolic acid family were the major fraction, specifically the chlorogenic acids and their related compounds. The most abundant compounds for all the analyzed coffees were chlorogenic acid and caffeine, with the highest values reported for coffee 6 (1168 ± 58 and 907 ± 45 mg/L, respectively). In samples 2, 7, 9, 10, and 12, caffeine was the most abundant phenolic compound. Meanwhile, chlorogenic acid was the most abundant compound in samples 1, 3–6, 8, and 11. However, this tendency was only observed in sample 1, since their major contributors to its phenolic compounds were neochlorogenic and protocatechuic acid, contributing at least 53% of the total amount. On the other hand, the *trans*-ferulic acid (0.55 ± 0.02 mg/L) in sample 1 and rutin in samples 5 and 12 (0.59 ± 0.02 and 0.56 ± 0.02 mg/L, respectively) were the phenolic compounds found in the lowest concentrations. 

Despite the similar phenolic profile from all the tested samples, some compounds stood out. For instance, the amounts of 3,5-di-caffeoylquinic and 3,4-di-O-caffeyolquinic acids in coffee 8 (99.6 ± 5.0 and 106 ± 5 mg/L, respectively) should be highlighted when compared with the other studied coffees. These values may result from the fact that sample 8 was the only ground coffee sample, while all the others were secured in capsules or formulated as instant coffee. The amount of hydroxybenzoic acid, namely the vanillic acid, quantified in coffees 2, 8–11, as well as the amounts of the flavanols catechin and epicatechin reported for coffees 3, 8, 10–12 were also notable; however, in this case, a correlation between the quantities of these compounds and the type of coffee was not observed.

The quantities of analyzed phenolics (summarized in Table S2) increased as follows: sample 1 < sample 10 < sample 9 < sample 7 < sample 12 < sample 4 < sample 3 < sample 5 < sample 2 < sample 11 < sample 8 < sample 6. 

Previous studies have characterized the phenolic profile of different coffee samples [42,43,44], and one of the published works also analyzed natural roasted coffee beans commercialized by the Portuguese company Delta Cafés [45]. Soares et al. [42] determined the phenolic profile of regular or decaffeinated espresso coffee, reporting that caffeine was the major contributor, with levels ranging from 2050 to 3265 mg/L, followed by caffeic acid (242.5 to 502.5 mg/L), chlorogenic acid (82.5 to 172.5 mg/L), ferulic acid (62.5 to 65.0 mg/L) and *p*-coumaric acid (52.5 to 70.0 mg/L). All the mentioned compounds were identified in the coffee samples in the present study, but their contents differed from those reported by these authors. Vilas-Boas et al. [44] also characterized the phenolic profile and antioxidant activity of 11 commercial expresso coffees prepared from a single-dose capsule system, reporting that caffeine was also the major contributor, with levels ranging from 2130 to 2790 mg/L. Again, these levels are higher than the amounts quantified in the present samples. This could be attributed to the different brands of coffees analyzed and the different expresso coffee preparations. In addition, Vilas-Boas et al. [44] focus on characterizing the chlorogenic acids composition, which are the main polyphenols present in coffee. These authors reported that chlorogenic acid (880 to 1120 mg/L), neochlorogenic acid (500 to 640 mg/L), and 4-caffeyolquinic acids (530 to 630 mg/L) were predominant, followed by 3,4- and 3,5-dicaffeyolquinic acids, with contents ranging from 30 to 80 mg/L. In the present study, the same pattern of chlorogenic acids composition was found, and similar quantities are also reported. 

As observed in Table S2, the 100 % Arabica coffees presented the most phenolic compounds. These results agree with the findings of Olechno et al. [31] and Hasbullah and Umiyati [30], but contradict the values obtained by Czachor et al. [13] and Vignoli et al. [35]. In Olechno et al. [31] the 100% Arabica coffee beverages presented marginally more phenolic compounds than the 100% Robusta coffee. Hasbullah and Umiyati [30] also verified that the total polyphenols in Arabica coffee were greater than in the Robusta coffee for all roast intensities. Nevertheless, in Czachor et al. [13] and Vignoli et al. [35] reported in independent studies that the TPC in Robusta coffee is significantly higher than that in Arabica. 

The different results obtained in these studies can be explained by the differences in the utilized coffee’s physicochemical composition, such as the coffee’s geographical origin, growth conditions, and processing. These lead to differences in the content of compounds in the green coffee beans and, ultimately, have an impact on the roasting process [13,15,30,32].

The coffee intensity is presumably related to the roasting degree [46] and can also explain some differences between samples. In this study, sample 4 has the highest roasting degree (13, very dark roast), and sample 8 has the lowest (5, light roast). Statistical analysis of the HPLC-DAD data for the different coffees identified few significant correlations between the compounds in different samples (Table S2). 

The analysis of the relationship between the OH^•^ and H_2_O_2_ scavenging activities and the polyphenolic composition of the coffee samples (Table S2) reveals a few interesting correlations. There is a mild to moderate positive correlation between OH^•^ and caftaric acid (r = 0.330); and H_2_O_2_ and the following compounds: protocatechuic acid (r = 0.419), neochlorogenic acid (r = 0.326), caffeine (r = 0.352), chlorogenic acid (r = 0.499) and rutin (r = 0.319). Conversely, some compounds exhibit a significant negative correlation with H_2_O_2_: vanillic acid (r = −0.554), p-coumaric acid (r = −0.578), kaempferol-3-O-glucoside (r = −0.619) and kaempferol-3-O-rutinoside (r = −0.534). Furthermore, compounds like gallic acid, syringic acid, (−)-epicatechin, and ellagic acid show a moderate negative correlation (r values between −0.5 and −0.3) with H_2_O_2_. Kaempferol-3-O-glucoside and kaempferol-3-O-rutinoside correlate similarly with OH^•^. Meanwhile, trans-ferulic acid, quercetin-3-O-galactoside, quercetin-3-O-glucopyranoside, and cinnamic acid exhibit this moderate negative correlation with both OH^•^ and H_2_O_2_.

According to the literature, the scavenging abilities of flavonols towards the radical OH^•^ are notably high, following the sequence: quercetin > myricetin > kaempferol [47]. Quercetin, which embodies all the essential structural features of a flavonol antioxidant—the 5-hydroxy-4-keto configuration in the A-ring, the 2,3-double bond which encourages ring conjugation, and the 3′,4′-dihydroxy catechol arrangement in the B-ring—stands out as a typical flavonoid [47]. It is expected to display superior antioxidant and antiradical actions [47]. In another study, gallic acid and pyrogallol demonstrated the most potent antioxidant and scavenging abilities of H_2_O_2_ and DPPH^•^ radicals [48]. These compounds have three hydroxyl groups in an ortho arrangement attached to their aromatic rings. Phenolic acids with two hydroxyl groups in the ortho position, like caffeic, protocatechuic, and o-pyrocatechuic acids, exhibited robust antioxidant and anti-radical properties as well [48]. α-Resorcylic and β-resorcylic acids, which have two hydroxyl groups in a meta-arrangement, showed average antioxidant capabilities and limited scavenging for DPPH^•^ and H_2_O_2_ [48]. Compounds with a single hydroxyl group displayed the least anti-radical and antioxidant potency [48]. The evidence indicates that the greater the number of hydroxyl groups attached to the aromatic ring, the stronger the antioxidant and anti-radical effects of the phenolic acids [48]. The low values of gallic (20.4 ± 1.0 mg/L), protocatechuic (179 ± 9 mg/L) and caffeic (11.4 ± 0.6 mg/L) acids in sample 1, could explain why this is the only sample with a TAC value for H_2_O_2_ lower than that for OH^•^. Regarding sample 7 that presents the lowest TAC values for OH^•^ and H_2_O_2_, it is probable that the lowest value of quercetin-3-O-glucopyranoside (1.83 ± 0.09 mg/L) and a low value of quercetin-3-O-galactoside (4.88 ± 0.24 mg/L) have the most important effect in these samples. Also, cinnamic acids, such as coumaric, caffeic, ferulic, and sinapic acids, demonstrate diverse biological properties such as neutralizing free radicals [49]. Sample 7 also has low values of these acids. However, it is essential to note that polyphenolic substances’ radical scavenging efficacy is not always consistent with the total antioxidant power determined by traditional techniques [47]. This discrepancy arises from the differences between kinetics (rate-driven) and thermodynamics (reaction proportion-driven) in radical scavenging and antioxidant functions, respectively [47]. 

Overall, the brewing technique can also have an impact on the total antioxidant capacity of coffee. Different coffee brewing methods employ varied extraction or infusion conditions, such as duration, temperature, pressure, and water-to-powder ratio. These conditions can impact the final coffee quality [50]. Gobbi et al. [50] sought to investigate the effects of four popular Italian coffee brewing techniques: the industrial espresso machine, Moka machine, pod machine, and the capsule machine. The study’s focus was on determining the quality and safety of the brewed coffee, considering factors like biogenic amines (BAs), total polyphenol content (TPC), total flavonoid content (TFC), and anti-radical activities (measured through DPPH and ABTS assays). These authors reported that capsule coffee preparations displayed the highest TPC and TFC values in the coffee powder. Yet, this pattern was not consistent during the infusion processes. Coffee infusions with the most concentrated total polyphenols in the final extract came from samples made with the Moka machine (2.71–3.52 mg GAE/g of coffee powder) and the professional bar machine (1.51–1.72 mg GAE/g of coffee powder), and for TFC, the Moka technique stood out as the most effective preparation method, yielding 8.55–8.60 mg RE/g of coffee powder. This might be attributed to the extended interaction between water and ground coffee and elevated extraction temperatures. The ratio of water volume to amount of coffee powder has been pinpointed as a constraining element in extracting TPC and TFC from coffee across all extraction methods [50]. Moreover, factors like temperature, the duration of water–coffee powder contact, pressure, and powder granularity can also influence polyphenol extraction from coffee in various extraction processes [50]. In our work, however, there were no evident relations between the brewing technique and the sample antioxidant activity or polyphenol content. 

## 4. Conclusions

The beneficial health effects of coffee are generally attributed to its antioxidant ability to inhibit the oxidation process, which can result in the formation of ROS. In this study, the antioxidant capacity of twelve local coffees available in Portuguese markets were assessed using conventional TAC assays: TPC, TFC, FRAP, DPPH and HPLC, and a electrochemical DNA-based biosensor constructed in our lab. 

Significant differences in the chemical compositions of coffee samples collected from the local markets were observed. When immersed with the HO^•^ radical, the antioxidant capacity for the DNA-based biosensor ranged from 164.95 to 1770.00 mg GAE/L, and from 193.43 to 2297.60 mg GAE/L in the presence of the H_2_O_2_ oxidant. Considering TPC, the values varied from 4932.15 to 9386.38 mg GAE/L, while for TFC, the values ranged from 2294.38 to 4117.84 mg ECE/L. The lowest and highest FRAP values were 3728.16 mg AAE/L and 11,205.26 mg AAE/L, respectively. In regard to the radical scavenging activity, the DPPH-RSA values ranged from 15,725 to 22,047 mg TE/L.

Regarding the phenolic composition, the HPLC analysis revealed that caffeine, chlorogenic acid, protocatechuic acid, neochlorogenic acid, and gallic acid were the main contributors to the phenolic profile of the twelve coffee samples and were possibly the main components responsible for the antioxidant capacity exhibited by the coffees. 

The DNA-based biosensor against the two ROS (HO^•^ and H_2_O_2_) was demonstrated to be a useful approach for TAC assessment as it is possible to simulate the protective effects of antioxidants on biological structures when exposed to the two analyzed radicals. The beverages prepared from blends composed of 100% Arabica coffee presented slightly higher protection against ROS. Significant differences (*p* > 0.05) were found between the coffee samples, even among those from the same brand. The daily consumption of coffee has been proven to provide a substantial portion of the daily intake of antioxidants.

## Figures and Tables

**Table 1:** Relevant information of the procured coffee samples for the antioxidant capacity assessment.

Sample	Presentation	Intensity (Degree of Roast)	Composition
1	Capsule	9	arabica + robusta
2	Capsule	10	arabica + robusta
3	Capsule (decaffeinated)	7	arabica + robusta
4	Capsule (power coffee)	13	blend coffee + guarana + ginseng
5	Capsule	10	arabica + robusta
6	Capsule	9	100% arabica
7	Capsule	8	arabica + robusta
8	Capsule	5	100% arabica
9	Capsule	7	arabica + robusta
10	Ground coffee	7	100% arabica
11	Capsule (decaffeinated)	9	100% arabica
12	Instant coffee	8	arabica + robusta

**Table 2:** Pearson correlations between optical and DNA-based biosensor methods.

	TPC	TFC	FRAP	DPPH	OH^•^	H_2_O_2_
**TPC**	1	0.5380 **	0.8140 **	−0.1900	−0.2970	0.1060
**TFC**	0.5380 **	1	0.3260	0.2230	−0.6150 **	0.0274
**FRAP**	0.8140 **	0.3260	1	−0.3710	−0.2250	−0.0095
**DPPH**	−0.1900	0.2230	−0.3710	1	−0.2750	−0.4180 *
**OH^•^**	−0.2970	−0.6150 **	−0.2250	−0.2750	1	0.4530 *
**H_2_O_2_**	0.1060	0.0274	−0.0095	−0.4180 *	0.4530 *	1

**. The correlation is significant at *p* < 0.01 (2-tailed); *. The correlation is significant at *p* < 0.05 (2-tailed).

## Data Availability

Not applicable.

## Supplementary Materials

The following supporting information can be downloaded at: https://www.mdpi.com/article/10.3390/bios13090840/s1, Table S1:Total antioxidant capacity of commercial coffee samples measured by a DNA-based biosensor and optical methods: Table S2: Phenolic compounds identified and quantified in coffee and coffee substitutes available in the Portuguese market; results, in mg/L of coffee, are expressed as mean ± standard deviation of three replicates.
