# Supplementary material for: Assessment of the Antioxidant Capacity of Commercial Coffee Using Conventional Optical and Chromatographic Methods and an Innovative Electrochemical DNA-Based Biosensor
Source: Biosensors (Basel). 2023 Aug 24;13(9):840. doi: 10.3390/bios13090840 (PMC10526474; doi:10.3390/bios13090840)
Supplement: Supplementary file 1 [file biosensors-13-00840-s001.zip › biosensors-2462924-supplementary.pdf]

Supplementary

Assessment of the Antioxidant Capacity of Commercial Coffee Using Conventional Optical and Chromatographic Methods and an Innovative Electrochemical DNA-Based Biosensor

Stephanie L. Morais [1], Diana Rede [1], Maria João Ramalhosa [1], Manuela Correia [1], Marlene Santos [2], Cristina Delerue-Matos [1], Manuela M. Moreira [1,*], Cristina Soares [1,*] and Maria Fátima Barroso [1,*]

[1] REQUIMTE/LAQV, Instituto Superior de Engenharia do Porto, Instituto Politécnico do Porto, Rua Dr. António Bernardino de Almeida 431, 4249-015 Porto, Portugal; stephanielopesmorais@gmail.com (S.L.M.); dsgmr@isep.ipp.pt (D.R.); mjr@isep.ipp.pt (M.J.R.); mmb@isep.ipp.pt (M.C.); cmm@isep.ipp.pt (C.D.-M.)

[2] Centro de Investigação em Saúde e Ambiente, Escola Superior de Saúde (CISA|ESS), Instituto Politécnico do Porto, Rua Dr. António Bernardino de Almeida 400, 4200-072 Porto, Portugal; mes@ess.ipp.pt

* Correspondence: manuela.moreira@graq.isep.ipp.pt (M.M.M.); cds@isep.ipp.pt (C.S.); mfb@isep.ipp.pt (M.F.B.)

Table 1S. Total antioxidant capacity of commercial coffee samples measured by a DNA-based biosensor and optical methods.

Sample	DNA-based biossensor		Optical methods			
	$OH^{\bullet}$ mg GAE/L	H_2O_2 mg GAE/L	TPC mg GAE/L	TFC mg ECE/L	FRAP mg AAE/L	DPPH mg TE/L
**1**	448.4 ± 12.1^{a,b,c}	227.9 ± 0.6^{a}	5611 ± 203^{a,b,c}	2566 ± 167^{a,b,c}	4468 ± 132^{a,b}	24154 ± 4719^{a,b}
**2**	939.4 ± 220.5^{b,d}	1562 ± 34^{b}	7343 ± 194^{b}	3280 ± 259^{b,d}	5062 ± 19^{a}	25811 ± 948^{a,b}
**3**	1771 ± 135^{e}	1824 ± 126^{b}	4612 ± 146^{a}	1940 ± 143^{a}	3493 ± 14^{c}	16990 ± 643^{a}
**4**	523.2 ± 52.2^{a,b,c}	1060 ± 58^{c}	6288 ± 760^{a,b,c}	3162 ± 211^{b,c,d}	4702 ± 68^{a}	29540 ± 4029^{b}
**5**	303.3 ± 75.3^{a,c}	1021 ± 65^{c,d}	5966 ± 133^{a,b,c}	3588 ± 76^{d}	4933 ± 56^{a}	21308 ± 5036^{a,b}
**6**	1275 ± 269^{d,e}	1709 ± 99^{b}	4670 ± 76^{a}	2300 ± 7^{a,c}	3683 ± 55^{b,c}	19964 ± 1843^{a,b}
**7**	155.9 ± 12.8^{a}	193.4 ± 10.8^{a}	5447 ± 98^{a,c}	2743 ± 114^{a,b,c,d}	4279 ± 85^{a,b,c}	22047 ± 473^{a,b}
**8**	454.5 ± 27.9^{a,b,c}	1187 ± 88^{c}	5787 ± 272^{a,b,c}	3304 ± 63^{b,d}	5161 ± 1^{a}	21942 ± 955^{a,b}
**9**	559.5 ± 38.6^{a,b,c}	2422 ± 20^{e}	11807 ± 995^{d}	3504 ± 670^{d}	9942 ± 548^{d}	15725 ± 969^{a}
**10**	425.3 ± 13.4^{a,c}	2297 ± 54^{e}	6449 ± 225^{b,c}	2909 ± 116^{b,c,d}	6248 ± 187^{e}	19837 ± 3343^{a,b}
**11**	322.2 ± 76.5^{a,c}	335.0 ± 71.4^{a}	10149 ± 719^{d,e}	3091 ± 123^{b,c,d}	8163 ± 293^{f}	24921 ± 2594^{a,b}
**12**	688.1 ± 200.5^{b,c}	699.4 ± 187.0^{d}	9296 ± 187^{e}	2894 ± 40^{b,c,d}	13805 ± 504^{g}	16954 ± 24^{a}

Table 2S. Phenolic compounds identified and quantified in coffee and coffee substitutes available in the Portuguese market; results, in mg/L of coffee, are expressed as mean ± standard deviation of three replicates.

Phenolic compound	Retention time / min	Coffee 1	Coffee 2	Coffee 3	Coffee 4	Coffee 5	Coffee 6	Coffee 7	Coffee 8	Coffee 9	Coffee 10	Coffee 11	Coffee 12
**Gallic acid**	5.618	20.4±1.0b	360±18a	42.5±2.1b,f	236±12d	61.1±3.1e,f,g	98.2±4.9c	71.2±3.6e,g	79.1±4.0c,e	51.8±2.6f,g	67.8±3.4e,f,g	102±5c	405±20h
**Protocatechuic acid**	9.935	179±9b	360±18a	528±26d	466±23c,d	651±33f	695±35f	347±17a,e	416±21c	285±14e	332±17a,e	460±23c	300±15a,e
**Neochlorogenic acid**	10.219	209±10b	474±24a	406±20c	396±20c	514±26a	618±31e	340±17c,d,f	458±23a,c	299±15d	332±17d,f	487±24a	385±19c,f
**(+)-Catechin**	14.143	19.9±1.0b	107±5a	45.5±2.3b,f	38.5±1.9b	67.4±3.4f,h	300±15c	235±12d,g	214±11d	161±8e	248±12g	302±15c	76.8±3.8h
**Caftaric acid**	15.436	ND	ND	ND	ND	ND	ND	11.1±0.6b	ND	ND	11.7±0.6b	14.0±0.7a	ND
**Caffeine**	16.154	46.3±2.3b	726±36a,e	643±32a,d	607±30d	842±42c	907±45c	692±35a,d	822±41c,e	637±32a,d	254±13f	838±42c	453±23g
**Chlorogenic acid**	17.869	71.6±3.6b	469±23a	769±38d	741±37d	1003±50g	1168±58f	566±28a	835±42c,d	319±16e	20.4±1.0b	885±44c	52.3±2.6b
**4-O-caffeyolquinic acid**	19.897	15.7±0.8a,g	40.9±2.0a	346±17b	387±19c	66.2±3.3d	485±24e	336±17c,e	541±27e	329±16b,f	363±18g	497±25c,e	451±23f
**Vanillic acid**	20.748	80.9±4.0a	524±26b	30.0±1.5c	1.52±0.08d	1.91±0.10c	2.47±0.12c	18.7±0.9c	337±17d	215±11e	251±13f	31.2±1.6c	241±12e,f
**Caffeic acid**	21.224	11.4±0.6b	41.3±2.1a	50.7±2.5e	98.0±4.9d	47.9±2.4e	61.0±3.1f	47.9±2.4c	90.2±4.5c	49.0±2.5d	61.0±3.1d	72.4±3.6g	9.84±0.49g
**Syringic acid**	22.283	<LOD	<LOQ	10.7±0.5b	<LOQ	ND	1.04±0.05d	5.25±0.26c	11.3±0.6a,b	5.37±0.27c	16.6±0.8e	11.9±0.6a	10.4±0.5b
**(-)-Epicatechin**	23.294	<LOD	<LOQ	113±6d	5.33±0.27b	13.2±0.7c	14.7±0.7c	15.8±0.8c	103±5a	14.9±0.7c	112±6d	101±5a	35.4±1.8e
**p-Coumaric acid**	33.758	<LOQ	7.02±0.35a	ND	ND	ND	ND	ND	6.67±0.33a	ND	6.53±0.33a	<LOQ	ND
**trans-Ferulic acid**	37.289	0.55±0.03b	4.09±0.20a	14.7±0.7e	1.90±0.09c,d	2.22±0.14c,d,f	11.0±0.6g	3.04±0.15f	4.07±0.20a	1.43±0.07b,d	10.7±0.5g	2.71±0.14c,f	3.86±0.19a
**Sinapic acid**	37.662	19.7±1.0b	68.0±3.4a	46.2±2.3c,d	46.7±2.3c,d	28.4±1.4e	41.3±2.1c	25.4±1.4b,e	49.7±2.5d	25.3±1.3b	29.1±1.5e	43.1±2.2c	8.28±0.41f
**trans-polydatin**	39.182	1.59±0.08b	11.2±0.6a	24.6±1.2d	13.4±0.7a	13.0±0.6a	40.4±2.0e	11.8±0.6a	18.3±0.9c	12.4±0.6a	26.8±1.3d	18.1±0.9c	7.28±0.36f
**3,5-di-caffeoylquinic acid**	50.127	<LOD	45.8±2.3a	ND	39.3±2.0b	ND	ND	ND	99.6±5.0c	17.9±0.9d	33.9±1.7b	38.7±1.9b	ND
**Quercetin-3-O-galactoside**	52.177	8.11±0.41b	27.6±1.4a	38.4±1.9f	23.5±1.2d	3.68±0.18c	33.4±1.7g	4.88±0.24c,e	7.14±0.36b,e	6.58±0.33b,e	19.9±1.0h	5.17±0.26c,e	4.85±0.24c,e

**Quercetin-3-O-glucopyranoside**	52.735	<LOD	14.3±0.7a	8.61±0.43e	13.0±0.6c	ND	6.65±0.33f	1.83±0.09b	4.57±0.23d	2.04±0.10b	ND	2.09±0.10b	ND
**Rutin**	53.284	0.71±0.04b,e	<LOD	1.21±0.06d	1.17±0.06d	0.59±0.03a	1.80±0.09g	0.99±0.05f	1.21±0.06d	0.80±0.04e	1.23±0.06d	1.46±0.07c	0.56±0.03a
**Phloridzin**	54.355	2.48±0.12a	ND	11.3±0.6e	4.40±0.22c	1.44±0.07d	9.14±0.46f	1.53±0.08d	ND	0.94±0.05d	8.57±0.43f	3.27±0.16b	1.60±0.08d
**Ellagic acid**	55.284	<LOQ	39.8±2.0a	21.3±1.1c	20.9±1.0c	ND	23.8±1.2d	ND	11.6±0.6b	ND	ND	13.0±0.65b	ND
**3,4-di-O-caffeyolquinic acid**	56.781	33.5±1.7b	65.3±3.3a	47.3±2.4c,f	32.3±1.6b	12.6±0.6e	58.4±2.9a,g	12.1±0.6e	106±5d	16.2±0.8e	16.4±0.8e	52.4±2.62c,g	42.3±2.1f
**Cinnamic acid**	58.47	4.10±0.21b	25.5±1.3a	19.8±1.0c	20.4±1.0c	1.46±0.07e	9.89±0.49f	0.66±0.03e	3.57±0.18b,d	1.66±0.08e	2.31±0.12d,e	<LOQ	4.60±0.23b
**Kaempferol-3-O-glucoside**	59.466	2.73±0.14b	15.8±0.8a	13.7±0.7c	<LOD	ND	4.67±0.23d	ND	ND	ND	8.64±0.43e	<LOD	ND
**Kaempferol-3-O-rutinoside**	60.277	<LOD	9.72±0.49a	9.20±0.46a	<LOQ	ND	5.71±0.29c	ND	ND	ND	7.47±0.37d	1.40±0.07b	ND
**Isorhamnetin-3-O-rutinoside**	61.568	4.75±0.24b	3.10±0.16a,c	3.28±0.16c	2.84±0.14a	ND	2.86±0.14a	ND	ND	ND	ND	ND	ND
**Σ Phenolic compounds**		733	3440	3244	3196	3332	4600	2478	4219	2450	2241	3983	2494

LOD, limit of detection. LOQ: limit of quantification; ND, not detected. Within line different letters indicate significant differences ($p < 0.05$) between samples.
